# Elimination of STH morbidity in Zimbabwe: Results of 6 years of deworming intervention for school-age children

**DOI:** 10.1371/journal.pntd.0008739

**Published:** 2020-10-23

**Authors:** Nicholas Midzi, Antonio Montresor, Masceline J. Mutsaka-Makuvaza, Claudio Fronterre, Portia Manangazira, Isaac Phiri, Olatunji Johnson, Gibson Mhlanga, Peter J. Diggle

**Affiliations:** 1 National Institute of Health Research, Ministry of Health and Child Care, Harare, Zimbabwe; 2 Department of Control of Neglected Tropical Diseases, World Health Organization, Geneva, Switzerland; 3 Centre for Health Informatics, Computing and Statistics, Lancaster University, Lancaster, United Kingdom; 4 Department of Epidemiology and Disease Control, Ministry of Health and Child Care, Zimbabwe; 5 Preventive Services, Ministry of Health and Child Care, Zimbabwe; Universidade Federal de Minas Gerais, BRAZIL

## Abstract

This paper reports the prevalence and intensity of soil-transmitted helminth (STH) infections measured in Zimbabwe before and after a control intervention based on annual deworming of school-age children (SAC) conducted from 2012 to 2018.

In 2010, epidemiological data were collected from 13 195 SAC in 255 randomly selected schools in all districts nationwide using, as diagnostic methods, the Kato–Katz and the formal ether stool concentration technique. At follow up, conducted in 2017, only Kato–Katz was performed; specimens were collected from 13 352 children in 336 schools. The data were evaluated using a geospatial approach. The national prevalence of STH infection in SAC was estimated at 5.8% at baseline, with 0.8% of infections of moderate and heavy intensity. Preventive chemotherapy (PC) targeted all 2.5 million children of school age enrolled in Zimbabwe, with coverage ranging from 49% to 85%. At follow up, national prevalence of STH in SAC was estimated at 0.8%; infections of moderate and heavy intensity almost disappeared (0.1% prevalence). As a result, Zimbabwe can suspend deworming activities in 54 districts and reduce the frequency of PC in the remaining six districts. The total amount of albendazole tablets needed will be approximately 100 000 a year.

## Author summary

We analysed the impact of 6 years of preventive chemotherapy intervention by the Ministry of Health and Child Care of Zimbabwe (MoHCC) to control STH infections in Zimbabwe.

We applied a geostatistical method to analyse the epidemiological data before and after the intervention. The results are interesting because they demonstrate a complete elimination of infection of moderate heavy intensity (that are considered the ones causing most of STH morbidity and a drastic reduction in prevalence that entails an estimated reduction of 90% in the need for anthelminthic medicines to maintain the situation and a consequent reduction in programme costs.

## Introduction

STH are intestinal parasites that are transmitted by soil contaminated with human excreta. The infections are caused by *Ascaris lumbricoides* (roundworms), *Trichuris trichiura* (whipworms), *Necator americanus* and *Ancylostoma duodenale* (hookworms). Each of the four STH species has distinct characteristics, but they are considered as a single group because of their similar transmission dynamics, diagnosis, and prevention and control measures [1].

More than 2 billion people are estimated to be infected with one or more STH species [2]. The damage to health caused by STH is proportional to the number of worms infecting the host. Infections of light intensity cause minimal associated morbidity, whereas infections of moderate to heavy intensity by large numbers of worms adversely affect nutritional status, impair cognitive processes and are estimated to cause the loss of an estimated 1.2 million disability-adjusted life-years (DALYs) in 2016 [3].

To achieve sustained control of STH infection and morbidity, the World Health Organization (WHO) recommends an approach that includes access to appropriate sanitation, hygiene education and periodic administration of anthelminthics, or preventive chemotherapy (PC), to populations at risk [1].

Globally in 2019, 96 countries needed PC for STH: 310 million preschool children and 762 million SAC [4].

WHO coordinates two large donation programmes of anthelminthics to control STH through PC in SAC: albendazole, donated by GlaxoSmithKline, and mebendazole, donated by Johnson & Johnson, totaling 600 million tablets/year [5]. The PC intervention is relatively simple: anthelminthics are distributed by teachers once or twice a year. Global coverage of PC steadily scaled up from 2010 to 2019 [5].

In 2010, a national survey conducted in all provinces of Zimbabwe assessed the baseline epidemiological situation of STH and schistosome infections in SAC. The survey was followed in 2012–2017 by six rounds of PC in the context of the national STH and schistosomiasis control programme in children and in 2018 by another survey to assess the impact of the intervention.

This paper reports the baseline STH situation, the PC interventions conducted, the impact of PC on STH prevalence and intensity of infection, and the resulting reduction in anthelminthics needed in Zimbabwe for STH control.

## Method

### Baseline study population

The baseline survey was conducted between September and October 2010 among children enrolled in the last three classes of primary schools, which were randomly selected in rural areas of the country, and was completed in metropolitan provinces (Harare, Bulawayo and Chitungwiza town) from July to August 2011. The national school-based survey was extended from 2010 to 2011 due to limited financial resources for the project in 2010 [6].

Primary-school children constitute the high-risk age group for STH in the community [7] and in countries such as Zimbabwe, which have very high school enrolment [8] school programmes constitutes a simple and low-cost intervention to reach virtually every school age child with health interventions and health education. The prevalence of STH in primary-school children is a proxy for the burden of the disease in the community [9]. This cost–effective tool could be used at district or national levels to provide information on community prevalence, aid decisions relating to treatment strategy and evaluate the numbers at risk.

### Sample size

The STH survey was integrated with one on schistosomiasis that was expected to have a prevalence in Zimbabwe of 37% [10]. We assumed the total number of primary-school children enrolled in Zimbabwe to be of 3 119 270 [11]. A sample size of 15 818 children was calculated using the EPI Info 6 statistical package to obtain results with a confidence level of 99% and a margin of error of 1%.

### Selection of schools for the survey

The number of schools selected in each district was calculated proportionally according to the district population and the calculated sample size. A random sampling method was used to select schools proportionally to size in each districts [12]. In each school, 50 children in the last three classes of primary school were randomly selected at a ratio of 1 male:1 female.

### Ethical issues and permission to conduct the study

The proposal to conduct a national STH and schistosomiasis survey in SAC was approved by the Medical Research Council of Zimbabwe, the Secretary for Health and Child Welfare and the Secretary for Education, Sport, Arts and Culture. The provincial medical and education directors also approved the programme and offered support during project implementation. Informed written parental consent and children’s assent was sought from study participants. Children were free to withdraw from the study at any time without prejudice.

### Distribution of consent forms for the baseline survey

One month ahead of the survey, letters explaining the aim of the survey and the consent forms were sent to all schools involved and distributed by the respective school heads to parents for children selected to participate in the survey. Parents were requested to sign the consent form only if they read and understood it. The teachers were requested to help the parents in case of difficulties in understanding the consent form, and school children were requested to provide oral consent.

### Specimen collection

Ten teams of experts were involved in the survey. Each team comprised the core team and the district team. The core team was made up of a team leader (laboratory expert from the National Institute for Health Research [NIHR]), the senior provincial laboratory expert, a technical assistant from NIHR and a driver. The district team comprised the Community Nurse or State Registered Nurse, the District Environmental Health Officer and the District Education Officer. The core team spent 30 successive days in the field (each deployed in one province); the district team worked with the core team only for the period when it was in their district. A single stool specimen was collected from each child and stored in a plastic container.

### Diagnosis of intestinal helminths

STH was diagnosed using two techniques: Kato–Katz [13] and formal ether stool concentration [14]. The Kato–Katz technique involves microscopic examination of a fixed quantity of faecal material to detect the presence and quantity of helminth eggs. A Kato–Katz template measuring 41.7 mg of stool was used in this study. Kato–Katz is generally preferred for diagnosis of intestinal helminths in the field because the egg counts (egg intensity) demonstrate not only infection but also provide an essential indirect measure of the morbidity caused by STH, which increases with increasing egg counts. However, the sensitivity of the technique reduces in light infections (< 100 eggs per gram/stool). To improve sensitivity the formal ether concentration technique, with approximately one gram of stool and concentrated eggs in stool by centrifugation, was concurrently used.

A single Kato–Katz thick smear (slide) was prepared from each individual and examined within 2 hours from preparation to detect and count hookworm and other STH ova while still visible. Another portion of the stool specimen was processed using the formol ether stool concentration technique and the slide was examined by microscopy for STH ova. The results from each technique individually were combined as follows: a person was considered negative for each STH species if no parasite ova were detected using both techniques. A person was considered positive for STH if ova were detected by either of the two techniques. Egg intensity was estimated using results from Kato–Katz only.

The results were calculated by province, by STH species and by prevalence of any STH.

## Preventive chemotherapy intervention

PC with single-dose albendazole (400 mg) was conducted annually (September–October) during 2012–2018 (six rounds) in all districts of the country targeting all SAC and pre-SAC, who were covered with deworming during child health days; adults were not targeted by this intervention. Some adults received albendazole in the context of the programme for the elimination of lymphatic filariasis Albendazole was administered in combination with praziquantel to control schistosomiasis.

### PC coverage

Every year from 2014, the MoHCC collected and reported data on the coverage of the intervention in schools in each district to WHO. These reports were analysed and the provincial coverage (number of SAC treated/number of SAC in the province) was calculated.

## Method used for impact assessment

**Fig 1:**
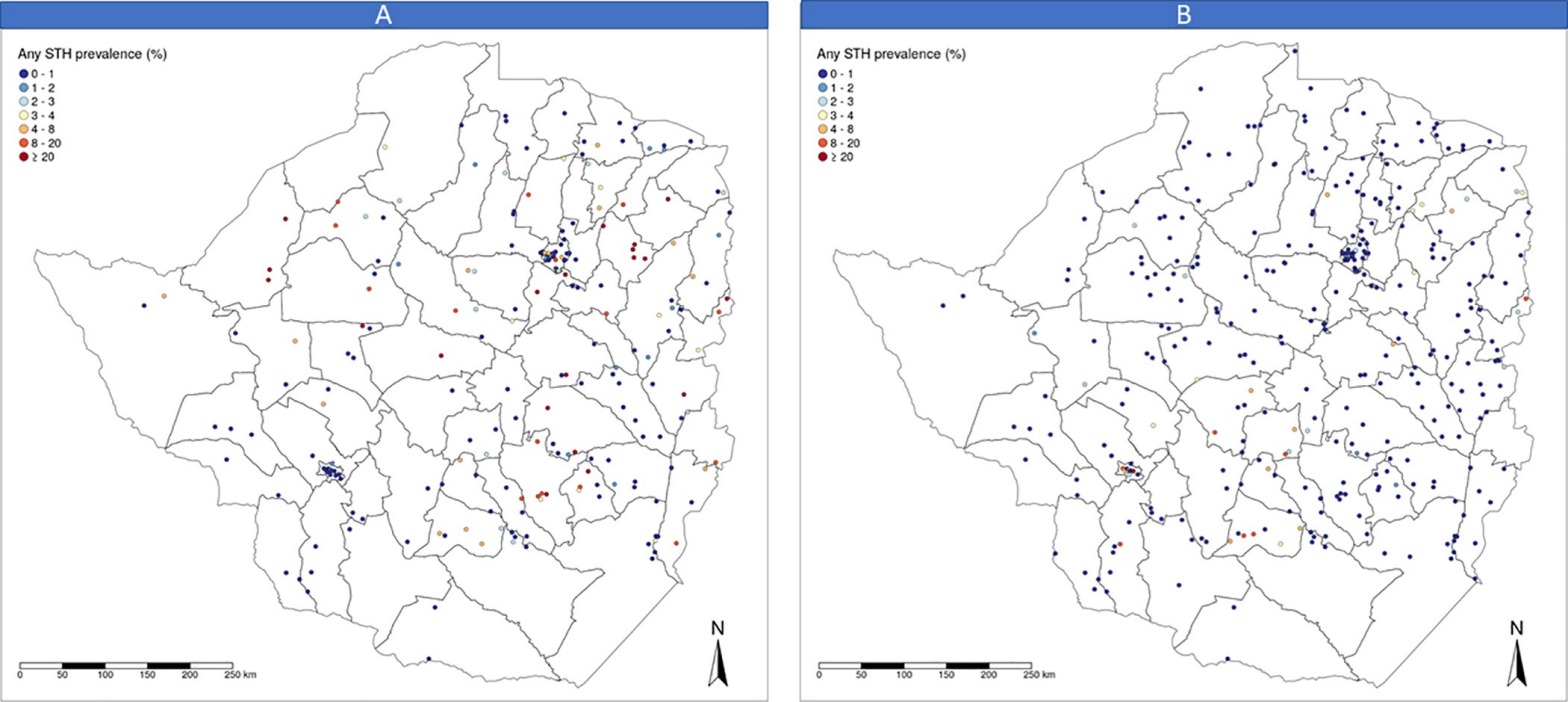
Maps presenting the location and STH prevalence in schools surveyed at baseline (A) 225 schools and at impact assessment survey (B) 336 schools.

The impact assessment survey was conducted between September and December 2018 with a method similar to that used in the baseline survey. The sample included all the schools investigated at baseline, with an additional 81 schools that were not involved in the baseline assessment, totalling 336 schools (see Fig 1).

Only the Kato–Katz laboratory method was used during the impact assessment, but this time using two slides prepared from a stool specimen collected from a single person on one day.

### Data analysis

Data were recorded on paper during microscope examination and transferred into an Excel file.

## Geospatial covariate selection and testing for spatial residual variation

Geospatial covariates are important to explain part of the variation observed in prevalence. First, we assembled a set of environmental, socioeconomic and physical covariates for the analysis including: proportion of open defecation in 2011 and 2015, elevation, enhanced vegetation index (EVI), normalized difference vegetation index (NDVI), rainfall, soil PH, soil moisture, composition of the soil, night light emission, land surface temperature for day and night and number of MDA rounds. These covariates were chosen because they are either associated with the prevalence of any of the STH parasites or serve as a proxy for other factors that are known to drive the prevalence of any of the STH parasites [15]. The complete list of covariates and their values in the binomial geostatistical model are provided in supplementary material S1 Table (baseline) and S2 Table (impact assessment).

We checked for multicollinearity and selected between environmental covariates with a Pearson correlation coefficient > 0.8 by looking at the empirical marginal relationship between the covariates and the empirical logit of STH prevalence.

In order to test for the presence of residual spatial variation, we fitted a non-spatial binomial regression model to the empirical prevalence including covariates. The residuals of the fitted model were examined for the presence of residual spatial variation not captured by the selected predictors. The empirical variogram of model residuals was compared with Monte Carlo based envelopes for the null hypothesis of no residual spatial correlation. If evidence of residual spatial correlation was found, we proceeded to fit a geostatistical model.

## Geostatistical modelling and prediction maps

The empirical prevalence data for each of the STH infections was modelled using a binomial logistic geospatial model by including two types of random effect in the linear predictor [16], the spatially correlated process and the spatially uncorrelated process. The former was included for two main reasons: to account for the geographical variation and, more importantly in this application, to predict prevalence at unobserved locations; the latter was included to account for the measurement error. More specifically, conditional on the random effects, we assumed that the numbers of pupils who tested positive for each of the STH infections at location *x_i_* were mutually independent binomial variables, with probability of being positive *P*(*x_i_*) such that $log(P\left(x_{i}\right)/\left(1-P(x_{i})\right))=d^{\prime }(x_{i})\beta +S(x_{i})+Z_{i}$, where *d*(*x_i_*) is the vector of covariate associated with associated coefficients *β*, *S*(*x*) is the latent, spatially continuous process that accounted for the unexplained residual spatial variation, and *Z_i_* is the Gaussian noise, to account for non-spatial extra-binomial variation. We employed Monte Carlo Maximum Likelihood (MCML) to estimate the model parameter as implemented in the PrevMap [17] R Package.

To carry out spatial prediction of STH, we entered the parameter estimates into the prediction equations described in the supplementary material. We predicted the prevalence of each STH parasite infection on a 5 km x 5 km regular grid throughout Zimbabwe. We then estimated the prevalence of any STH using the equation described in de Silva and Hall [18] given as:

$$P_{any}=\frac{\left(a+t+h)-(a\times t+a\times h+t\times h)+(a\times t\times h\right)}{1.06},$$

where *a, t* and *h* are the prevalence of ascariasis, trichuriasis and hookworm infection, respectively.

Additional details on geostatistical modelling are presented in supplementary material S1 Text; additional details on inference an are presented in supplementary material S2 Text and additional details on prediction map are presented in supplementary material S3 Text.

Instead of producing the map of the mean and the standard error of STH prevalence, we mapped the probability that the STH parasite infections and any STH prevalence would not exceed the WHO threshold for treatment (i.e. 20% at baseline and 2% after 5 years of intervention).

The thresholds are different at the different time points of the control programme because the STH prevalence at baseline tends to remain stable (because it results from an equilibrium among parasite, host and environment). Conversely, after several years of drug administration the STH prevalence tends to return to the original equilibrium when drug intervention is suspended.

For this reason, and to prevent losing the advantages gained with the drug intervention, the threshold for the provision of drug intervention is much lower at follow up than at baseline (i.e. 2% versus 20%).

All data analyses including the mapping were carried out in R statistical software. Further detailed information on the fitted model is provided in the supplementary material.

Because deworming is extremely low cost, we decided to be restrictive in excluding districts from intervention: we excluded a district from intervention when the probability of STH prevalence to be under the WHO threshold was estimated at > 70%.

## Results

### Baseline

Estimates of parameters of the fitted models are shown in the supplementary material. At baseline, 13 195 children enrolled in 255 primary schools were investigated. The mean age of the school children in the sample was of 11.2 years (range 10–15); girls made up 50.7% of the total.

Fig 1A shows the location of the schools surveyed and the empirical prevalence of any STH.

Prevalence of STH infections in SAC was estimated nationally at 5.8% and the prevalence of infection of moderate or heavy intensity at 0.8%. Table 1 shows details of the prevalence of STH by province and by STH species.

**Table 1:** Prevalence of STH and prevalence of moderate and heavy intensity STH infection (MHI) by species by province at baseline survey (2010–2011) and at impact survey (2018–2019).

Province	Any STH			*Ascaris lumbricoides*			*Trichuris trichiura*		Hookworms	
	Baseline		Impact survey	Baseline.		Impact survey	Baseline	Impact survey	Baseline	Impact survey
	Prevalence (CI)	Prevalence MHI (CI)	Prevalence (CI)	Prevalence MHI (CI)	Prevalence (CI)	Prevalence (CI)	Prevalence (CI)	Prevalence (CI)	Prevalence (CI)	Prevalence (CI)
Manicaland	3.8 (3.0, 4.7)	1.7 (1.1, 2.2)	0.9 (0.5, 1.3)	0.3 (0.1, 0.5)	1.7 (1.1, 2.3)	0.4 (0.2, 0.7)	0.3 (0.1, 0.6)	0.1 (0.3, 0.6)	2.6 (1.9, 3.3)	0.3 (0.1, 0.5)
Mashonaland East	19.9 (17.4, 22.3)	2.5 (1.6, 3.3)	1.0 (0.4, 1.5)	0.1 (0.0, 0.2)	19.6 (17.1, 22.0)	1.0 (0.4, 1.5)	0.1 (0.0, 0.3)	0.0 (0.0, 0.0)	1.1 (0.4, 1.7)	0.0 (0.0, 0.0)
Mashonaland Central	1.8 (0.9, 2.6)	0.3 (0.0, 0.6)	0.6 (0.2, 0.9)	0.1 (0.0, 0.3)	0.9 (0.3, 1.5)	0.1 (0.0, 0.2)	0.4 (0.0, 0.8)	0.1 (0.0, 0.2)	0.6 (0.1, 1.1)	0.6 (0.2, 0.9)
Mashonaland West	3.8 (2.3, 5.3)	0.3 (0.0, 0.7)	0.0 (0.0, 0.0)	0.0 (0.0, 0.0)	1.3 (0.4, 2.2)	0.0 (0.0, 0.0)	0.0 (0.0, 0.0)	0.0 (0.0, 0.0)	2.6 (1.4, 3.9)	0.0 (0.0, 0.0)
Masvingo	6.3 (5.1, 7.6)	0.5 (0.2, 0.8)	0.1 (0.0, 0.2)	0.1 (0.0, 0.2)	0.1 (0.0, 0.2)	0.1 (0.0, 0.2)	0.1 (0.0, 0.2)	0.1 (0.0, 0.2)	6.3 (5.1, 7.6)	0.0 (0.0, 0.0)
Matabeleland North	16.0 (13.5, 18.5)	0.0 (0.0, 0.0)	0.6 (0.1, 1.1)	0.0 (0.0, 0.0)	0.0 (0.0, 0.0)	0.4 (0.0, 0.8)	0.0 (0.0, 0.0)	0.0 (0.0, 0.0)	16.0 (13.5, 18.5)	0.2 (0.0, 0.6)
Matabeleland South	0.0 (0.0, 0.0)	0.0 (0.0, 0.0)	0.6 (0.1, 1.0)	0.0 (0.0, 0.0)	0.0 (0.0, 0.0)	0.0 (0.0, 0.0)	0.0 (0.0, 0.0)	0.0 (0.0, 0.0)	0.0 (0.0, 0.0)	0.6 (0.1, 1.0)
Midlands	2.5 (1.5, 3.6)	0.4 (0.0, 0.9)	2.0 (1.3, 2.7)	0.0 (0.0, 0.0)	0.2 (0.0, 0.5)	1.8 (1.1, 2.5)	0.0 (0.0, 0.0)	0.0 (0.0, 0.0)	2.4 (1.4, 3.4)	0.0 (0.2, 0.4)
Harare	1.6 (0.7, 2.5)	0.3 (0.0, 0.7)	0.4 (0.0, 0.9)	0.0 (0.0, 0.0)	0.2 (0.0, 0.6)	0.4 (0.0, 0.9)	0.0 (0.0, 0.0)	0.0 (0.0, 0.0)	1.4 (0.6, 2.2)	0.0 (0.0, 0.0)
Bulawayo	0.3 (0.0, 0.7)	0.2 (0.0, 0.6)	4.3 (1.6, 7.1)	0.0 (0.0, 0.0)	0.3 (0.0, 0.7)	4.3 (1.6, 7.1)	0.0 (0.0, 0.0)	0.0 (0.0, 0.0)	0.0 (0.0, 0.0)	0.0 (0.0, 0.0)
Chitungwiza	2.4 (0.5, 4.3)	0.9 (0.0, 2.2)	0.7 (0.0, 2.1)	0.0 (0.0, 0.0)	1.6 (0.0, 3.2)	0.7 (0.0, 2.1)	0.0 (0.0, 0.0)	0.0 (0.0, 0.0)	1.2 (0.0, 2.5)	0.0 (0.0, 0.0)
Overall	**5.8** **(5.4, 6.3)**	**0.8** **(0.6, 0.9)**	**0.8** **(0.6, 0.9)**	**0.1** **(0.0, 0.1)**	**2.6** **(2.3, 2.9)**	**0.5** **(0.4, 0.7)**	**0.1** **(0.1, 0.2)**	**0.1** **(0.0, 0.1)**	**3.4** **(3.1, 3.8)**	**0.2** **(0.1, 0.3)**

**Fig 2:**
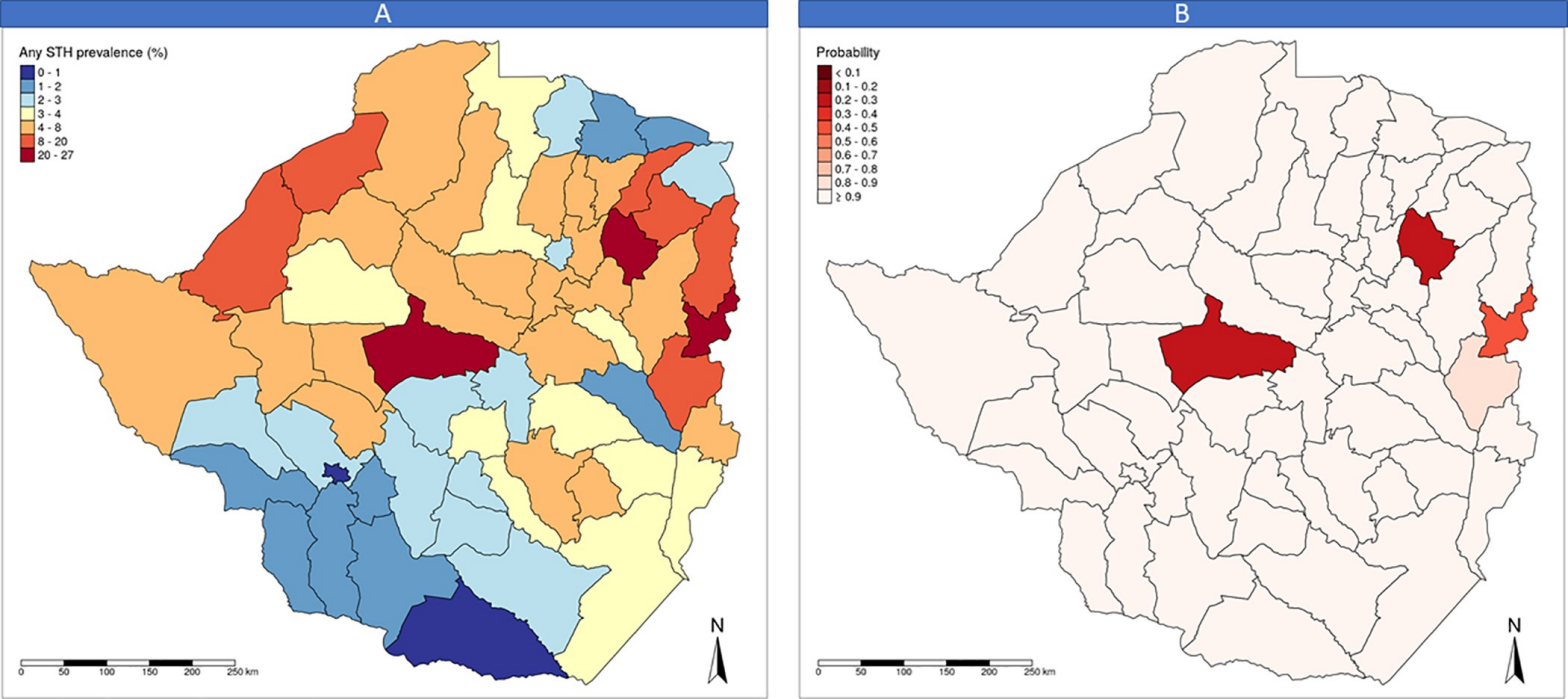
Maps presenting the results of baseline survey: (A) Predicted STH prevalence at baseline by district and (B) probability of a district being under the WHO threshold for intervention.

Fig 2A shows the predicted mean prevalence at baseline by district and Fig 2B shows the probability for a district to be under the WHO prevalence threshold for intervention at baseline (i.e. 20% prevalence corresponding to annual treatment) [7]. According to the WHO threshold, all districts excluding three (Kwekwe, Murehwa and Mutasa) had > 70% probability to be under the WHO threshold; therefore, only the three districts deserved treatment according to the WHO recommendation [7]. The MoHCC also evaluated, in addition to these epidemiological results, the cost–benefit of the intervention to select the control activities: Given the planned distribution of praziquantel to all children in the country and considering that the addition of albendazole (a donated medicine) to this intervention would have resulted only in marginal additional costs, the MoHCC decided to cover all the children with regular deworming.

### Intervention

A total of six PC rounds were organized between 2012 and 2017 covering the entire country, and over 16 million tablets of albendazole were donated to Zimbabwe through WHO for this purpose during the 6 years of intervention. The estimated national coverage of SAC during the 6 years ranged from 40% to 79%; Table 2 presents the coverage of the intervention in the different provinces by year. The coverage in the 6 year intervention has been on average of 51%, and has been on average lower on urban areas (Harare, Chitungwiza and Bulawayo = 30%) than rural areas (56%). In 2016 the national coverage has been particularly high (79%) because of the improved coverage in both urban (coverage 84%) and urban areas (coverage 43%).

**Table 2:** Coverage of deworming of school-age children in Zimbabwe by province and by year.

Province	Target population*	Year**					Average coverage during the 5 years programme
		2013	2014	2015	2016	2017	
Manicaland	181,789	25%	58%	60%	99%	60%	60%
Mashonaland East	528,337	51%	50%	52%	87%	52%	58%
Mashonaland Central	676,813	55%	40%	56%	70%	57%	56%
Mashonaland West	1,033,606	32%	36%	38%	86%	38%	46%
Masvingo	461,860	50%	48%	67§	93%	67%	65%
Matabeleland North	320,491	64%	38%	62%	93%	62%	64%
Matabeleland South	458,389	Not targeted	72%	Not targeted	77%	69%	73%
Midlands	486,760	51%	40%	39%	85%	39%	51%
Harare	617,956	28%	21%	26%	49%	26%	30%
Bulawayo	252,518	18%	10%	45%	14%	45%	26%
Chitungwiza	137,920	40%	21%	40%	70%	40%	42%
Overall	5,272,043	**40%**	**41%**	**47%**	**79%**	**47%**	**51%**

* Targeted population in 2014 (in the following year the population slightly increased)

** 2012 coverage data not available.

### Impact assessment

During impact assessment, 13 352 children enrolled in 336 primary schools were investigated. The mean age of the school children in the sample was 11.4 years (range 10–15); the girls made up 54.1% of the sample.

Fig 1B shows the location of the schools surveyed during the impact assessment survey and the prevalence of any STH measured. At impact evaluation survey, the prevalence of STH infections in SAC was estimated nationally at 0.8% and the prevalence of infection of moderate or heavy intensity at 0.1%. Table 1 shows details of the prevalence of STH by province and species.

**Fig 3:**
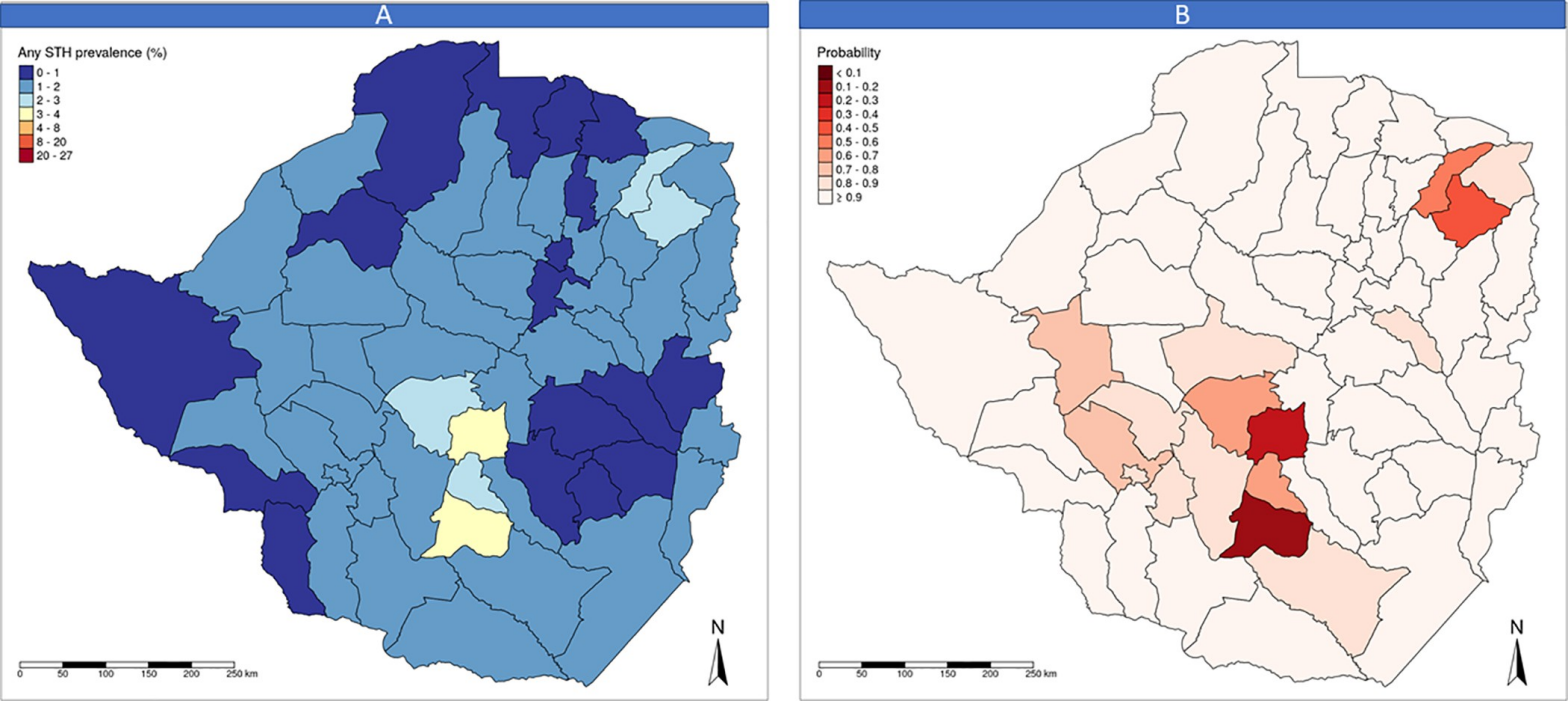
Maps presenting the results of Impact assessment survey: (A) predicted STH prevalence at baseline by district and (B) Probability of a district being under the WHO threshold for intervention.

Fig 3A shows the predicted STH prevalence estimated with impact evaluation survey and Fig 3B shows the probability of a district being under the WHO prevalence threshold for intervention at follow up (i.e. 2% corresponding to one treatment every 2 years) [7]. According to this evaluation, all districts except six (Gweru, Mberengwa, Mutoko, Shurugwi, UMP and Zvishavane) had a > 70% probability of prevalence being under < 2% and all districts a > 98% probability of STH prevalence being < 10%. Deworming intervention once every 2 years is therefore recommended in the six districts mentioned.

## Discussion

The national control programme in schools has been successful in reducing STH prevalence and in virtually eliminating STH infections of moderate and heavy intensity. Interestingly, the province in which the STH prevalence remained relatively high (i.e. Bulawayo, with STH prevalence estimated at 4.3%) is also the province in which the coverage the intervention was low (26%). Of interest also is that the coverage of the intervention was constantly lower in urban areas than in rural provinces. We were not able to investigate the specific reasons for this difference in Zimbabwe but lower coverage if PC campaigns is a constant observation and the reasons have been attributed to the higher heterogeneity and mobility of the urban population, reduced trust on public health services and weak public health infrastructure [19,20].It is remarkable that, while in districts with a relatively high prevalence at baseline (i.e. > 10%), the 6 years of intervention yielded a consistent and significant reduction of prevalence levels (i.e. from an average of 18% to an average of 1.4%, corresponding to a reduction of > 90%). In districts where the prevalence was already low at baseline (i.e. < 3%), the reduction was much less significant (from 1.8% to 1.3%, corresponding to a 27% reduction). In addition, in 25% of those districts the prevalence at impact evaluation survey was slightly higher than at baseline, which probably indicates the poor sensitivity of the diagnostic method used (i.e. Kato–Katz), at very low prevalence, but also the fact that in areas where the environment is contaminated it is probably impossible to reduce STH to below a certain level with drug intervention only; improvements in sanitation will be essential to eliminate STH completely in Zimbabwe and worldwide [1,5,21].

### Selection of the appropriate intervention in Zimbabwe

After the evaluation of the baseline survey conducted in 2010, the MoHCC decided to provide a more intensive control intervention for STH than that suggested by WHO.

According to map (b) in Fig 2, only three districts would have qualified for annual distribution of anthelminthics. However, because an intervention to control schistosomiasis targeting all SAC was also planned in Zimbabwe and because adding another anthelminthic to the distribution would have resulted only in marginal additional costs, the MoHCC decided to provide treatment for STH to all SAC in the country.

Adaptation of the WHO recommendations to the local situation is a prerogative of national health ministries. In Zimbabwe, the opportunity to distribute anthelminthics at very low additional cost was a strong justification to intensify control of STH.

### Impact of STH morbidity

Infections of moderate and heavy intensity were rare at baseline (0.8%) and were further reduced at impact evaluation survey (0.1%). In addition, after the PC intervention, in most provinces (7/11) no infection of moderate or heavy intensity was found and in no district the confidence interval of prevalence of infection of moderate and heavy intensity was over 2% (the threshold set by WHO for evaluate the elimination of STH morbidity in 2030) [21]. Additionally, the prevalence of STH infection of light intensity is low (< 1% at national level). We can therefore conclude that STH morbidity has been totally or almost completely eliminated in SAC in Zimbabwe.

### Reduction of frequency of drug administration

After the 6 years of intervention, the impact survey identified six districts in Zimbabwe that qualify for PC intervention once every 2 years [7]. Despite the marginal cost of the school deworming intervention, the MoHCC, having considered the elimination of morbidity obtained in the country and the fact that anthelminthics are needed in other countries where STH are still not under control, recommended to reduce the frequency of PC and to limit it only once every 2 years in the six districts identified. This reduction in the extension and frequency of PC in the country is expected to reduce the need for anthelminthics in Zimbabwe to approximately 212 000 tablets every 2 years, instead of the average 2.6 million per year requested from 2012 to 2017.

### Limitations of the study

The evaluation of the impact of the intervention on STH was part of the routine monitoring activities managed by the MoHCC and does not have the rigor of a scientific study: at baseline, a single specimen was collected from each child and a single Kato–Katz slide was prepared, while at follow up two slides from each specimen were prepared. This could have resulted in an underestimation of the impact of the intervention.

We did not measure directly the morbidity that is caused by STH, but assumed that when STH infection of MHI are not present in the population, STH morbidity has been eliminated. This assumption is related to the fact that STH infections with few worms (light intensity infections) cause minimal morbidity, whereas infections with many worms (moderate and heavy intensity infections) cause severe or clinical morbidity [1]; both WHO [22] and the Global Burden of Disease Study [23] use this indicator to evaluate STH morbidity.

An additional limitation of our study is that we did not included the diagnosis of *Strongyloides stercoralis* in our survey and do not implement control measures targeting this parasite. This is due to the fact that in Zimbabwe, as in all other STH-endemic countries, control of *S*. *stercoralis* in not part of STH control activities, mainly as a consequence of the difficulties in procuring ivermectin at an affordable price [24]. Merck Sharp & Dohme (MSD) restricts the use of donated ivermectin for treatment of onchocerciasis and lymphatic filariasis only.

### Results in context

The results obtained by the STH control programme in Zimbabwe confirm that is possible to reach an extremely low level of STH prevalence and to totally or almost completely eliminate morbidity due to STH. However, a level of sanitation that impedes environmental contamination with human faeces is necessary to interrupt transmission [1].

## Supporting information

S1 Table Parameter estimates of the binomial geostatistical model for the baseline data. (DOCX)

S2 Table Parameter estimates of the binomial geostatistical model for the impact data. (DOCX)

S3 Table STH prevalence at baseline and follow up by district. (XLSX)

S4 Table Coverage of Preventive Chemotherapy for STH in Zimbabwe 2013–2017. (XLSX)

S1 Text Approach for geostatistical modelling. (DOCX)

S2 Text Approach for inference estimation. (DOCX)

S3 Text Approach for the development prediction maps. (DOCX)
