# Supplementary material for: Elimination of STH morbidity in Zimbabwe: Results of 6 years of deworming intervention for school-age children
Source: PLoS Negl Trop Dis. 2020 Oct 23;14(10):e0008739. doi: 10.1371/journal.pntd.0008739 (PMC7641467; doi:10.1371/journal.pntd.0008739)
Supplement: S1 Table — (DOCX) [file pntd.0008739.s001.docx]

**S.1 Table:** Parameter estimates of the binomial geostatistical model for the baseline data

| Parameter | *Ascaris lumbricoides* | *Trichuris trichiura* | Hookworms |
| --- | --- | --- | --- |
| Intercept | -6.074  (-30.891, 18.744) | -7.752  (-9.191, -6.831) | -10.336  (-27.877, 7.206) |
| proportion of open defecation | -0.019  (-0.039, 0.002) | -0.408  (-1.622, 0.640) | 0.035  (0.021, 0.050) |
| Elevation | -0.001  (-0.004, 0.002) | -0.883  (-2.538, 0.655) | -0.001  (-0.003, 0.001) |
| night land surface temperature for day | -0.301  (-0.685, 0.084) | NA | 0.050  (-0.261, 0.362) |
| NDVI | 0.383  (-9.126, 9.893) | 0.851  (-0.308, 2.084) | 1.023  (-5.575, 7.621) |
| night light emission | -0.009  (-0.045, 0.028) | NA | 0.027  (-0.008, 0.062) |
| rainfall | -0.007  (-0.051, 0.037) | -0.100  (-0.792, 0.641) | 0.063  (0.035, 0.092) |
| sand | 0.008  (-0.050, 0.066) | 0.843  (-0.777, 2.943) | -0.003  (-0.047, 0.040) |
| soil moisture | 0.075  (0.036, 0.114) | 0.615  (-0.595, 1.798) | 0.021  (-0.001, 0.042) |
| soil PH | 1.436  (-0.707, 3.578) | -0.543  (-2.868, 1.583 | -0.156  (-1.819, 1.508 |
| $\sigma^{2}$ | 3.911  (2.780, 5.041) | NA | 1.651  (1.152, 2.151) |
| $\phi$ | 31.161  (18.078, 44.245) | NA | 6.989  (2.570, 11.408) |
| $\tau^{2}$ | 0.004  (-0.017, 0.024) | NA | 0.101  (-0.114, 0.316) |

Note: NA corresponds to a situation when the term is not included in the model.
