# Supplementary material for: Elimination of STH morbidity in Zimbabwe: Results of 6 years of deworming intervention for school-age children
Source: PLoS Negl Trop Dis. 2020 Oct 23;14(10):e0008739. doi: 10.1371/journal.pntd.0008739 (PMC7641467; doi:10.1371/journal.pntd.0008739)
Supplement: S2 Table — (DOCX) [file pntd.0008739.s002.docx]

**S.2 Table:** Parameter estimates of the binomial geostatistical model for the impact data

| Parameter | *Ascaris lumbricoides* | *Trichuris trichiura* | Hookworms |
| --- | --- | --- | --- |
| Intercept | -10.336  (-27.877, 7.206) | -7.356  (-8.140, -6.735) | -2.475  (-4.735 -0.216) |
| proportion of open defecation | 0.035  (0.021, 0.050) | NA | NA |
| Elevation | -0.001  (-0.003, 0.001) | NA | 3.457  (1.488, 5.425) |
| night land surface temperature for day | 0.050  (-0.261, 0.362) | NA | -0.038  (-0.103 0.027) |
| NDVI | 1.023  (-5.575, 7.621) | NA | -2.070  (-4.081, -0.059) |
| night light emission | 0.027  (-0.008, 0.062) | NA | -0.072  (-0.120, -0.025) |
| rainfall | 0.063  (0.035, 0.092) | NA | NA |
| sand | -0.003  (-0.047, 0.040) | NA | -0.016  (-0.026, -0.006) |
| soil moisture | 0.021  (-0.001, 0.042) | NA | -0.008  (-0.013, -0.003) |
| soil PH | -0.156  (-1.819, 1.508) | NA | NA |
| $\sigma^{2}$ | 1.651  (1.152, 2.151) | NA | 0.096  (0.012, 0.180) |
| $\phi$ | 6.989  (2.570, 11.408) | NA | 5.151  (-1.109, 11.411) |
| $\tau^{2}$ | 0.101  (-0.114, 0.316) | NA | 0.916  (-0.717, 2.549) |
| Elevation^2 | NA | NA | 0.430  (-0.622, 1.483) |
| NDVI^2 | NA | NA | 2.325  (1.366 3.285) |
| Number of MDA rounds (1) | NA | NA | 0.274  (0.132, 0.415) |
| Number of MDA rounds (2) | NA | NA | 0.177  (-0.002, 0.357) |

Note: NA corresponds to a situation when the term is not included in the model.
