# Supplementary material for: Elimination of STH morbidity in Zimbabwe: Results of 6 years of deworming intervention for school-age children
Source: PLoS Negl Trop Dis. 2020 Oct 23;14(10):e0008739. doi: 10.1371/journal.pntd.0008739 (PMC7641467; doi:10.1371/journal.pntd.0008739)
Supplement: S1 Text — (DOCX) [file pntd.0008739.s005.docx]

**S.3 Text:** Approaches for geostatistical Modelling

The variation in the prevalence of STH can be captured by a set of relevant environmental variables (listed in the paper). Having adjusted for these environmental variables, the remaining variation, unexplained component can therefore be captured by including a latent, spatially correlated process in the linear predictor. Let $Y_{i}$denote the number of pupils tested positive for STH out of $m_{i}$sampled pupils at school location $x_{i}:i=1,...n$. Let $S\left( x \right)$ denote a latent, spatially continuous process usually used to account for the unexplained residual spatial variation and $Z_{i}$denote the non-spatial residual variation usually used to capture the measurement error. We assume that conditional on $S\left( x \right)$, $Y_{i}$is independent and binomially independent random variables with binomial probabilities $P\left( x_{i} \right)$with denominator $m_{i}$. Therefore, the model for $P\left( x_{i} \right)$, the prevalence of STH at location $x_{i}$is given by

$log\left( P\left( x_{i} \right)/\left( 1-P\left( x_{i} \right) \right) \right)=d^{'}\left( x_{i} \right)\beta+S\left( x_{i} \right)+Z_{i}$, (1)

where $d\left( x_{i} \right)$is a vector of covariate associated with associated coefficient $\beta$. We specify $S\left( x \right)$has a Gaussian process with covariance function

$Cov\left\{ S\left( x \right),S\left( x' \right) \right\}=\sigma^{2}\rho\left( u;\theta\right),$

where $u=\left\| x-x' \right\|$is the ecludian distance between observation at location $x$and $x'$, $\sigma^{2}$is the variance of the Gaussian process and $\rho\left( u;\theta\right)$is a correlation function usually choosen from the Matern family indexed by parameter $\theta$. Modelling $S\left( x \right)$in this manner serves two purposes, to acount for the geographical variation and more importantly in this application, to give prediction of prevalences at unobserved locations. $Z_{i}$is modelled as a Gaussian noise with variance parameter $\tau^{2}$.

We developed the model described above to each specie of STH infection and we generate the prevalence of any STH using

$P_{any}=\frac{\left( a+t+h \right)-\left( a\times t+a\times h+t\times h \right)+\left( a\times t\times h \right)}{1.06}.$
