# Supplementary material for: Elimination of STH morbidity in Zimbabwe: Results of 6 years of deworming intervention for school-age children
Source: PLoS Negl Trop Dis. 2020 Oct 23;14(10):e0008739. doi: 10.1371/journal.pntd.0008739 (PMC7641467; doi:10.1371/journal.pntd.0008739)
Supplement: S2 Text — (DOCX) [file pntd.0008739.s006.docx]

**S 4 Text:** Approaches for inference estimation

We carry out parameter estimation using Monte Carlo Maximum Likelihood, implemented in PrevMap, an R package for analysing prevalence data, freely available from the Comprehensive R Archive Network (www.r-project.rg).
