# Supplementary material for: Elimination of STH morbidity in Zimbabwe: Results of 6 years of deworming intervention for school-age children
Source: PLoS Negl Trop Dis. 2020 Oct 23;14(10):e0008739. doi: 10.1371/journal.pntd.0008739 (PMC7641467; doi:10.1371/journal.pntd.0008739)
Supplement: S3 Text — (DOCX) [file pntd.0008739.s007.docx]

**S.5 Text:** Approach for development of prediction maps

To carry out spatial prediction, we plug the parameter estimates of the model described above into the prediction equations. We predict the STH prevalence on a 5 by 5 km regular grid over the whole region of Zimbabwe. The target of prediction is now given by the linear predictor at the $q$ prediction locations $x_{n+1},...,x_{n+q},$ defined as $\eta_{n+i}=d^{'}\left( x_{n+i} \right)\beta+S\left( x_{n+i} \right)$. Let $\eta^{\text{*}}=\left( \eta_{n+1},...,\eta_{n+q} \right)$denote the vector of the linear predictor values at prediction locations $x_{n+i}$. The answer to our prediction problem is given by the conditional distribution of $\eta^{\text{*}}$ given $y$, given as

$f\left( \eta^{\text{*}}\vee y \right)=\int f\left( \eta^{\text{*}}\vee\eta\right)f\left( \eta\vee y \right)d\eta$

where we used the fact that $\eta^{\text{*}}$ and $y$ are independent conditionally on $\eta$. The above equation allows us to carry out spatial prediction using the following approach: simulate samples from the conditional distribution of $\eta$ given $y$; using samples from the previous step, simulate samples from . The samples of $\eta^{\text{*}}$ obtained using this simulation procedure are then drawn from $f\left( \eta^{\text{*}}\vee y \right)$. The prediction of STH prevalence at location $x_{n+i}$is given by

$p\left( x_{n+i} \right)=\frac{e^{\eta^{\text{*}}}}{1+e^{\eta^{\text{*}}}}.$

Since in our application it is also of interest to identify locations with prevalence below a particular threshold, say $l$ , we summarise the predictive distribution of prevalence using non-exceedance probabilities (NEP). These are computed from the N samples from the distribution of $\eta^{\text{*}}$given $y$as

$NEP=\frac{1}{N}\sum I\left( \frac{e^{\eta^{\text{*}}}}{1+e^{\eta^{\text{*}}}}<l \right).$
